# Higher *In vitro* Proliferation Rate of *Rhizopus oryzae* in Blood of Diabetic Individuals in Chronic Glycaemic Control Compared with Non-diabetic Individuals

**DOI:** 10.1007/s11046-017-0174-0

**Published:** 2017-07-06

**Authors:** Grace Salazar-Tamayo, Luis E. López-Jácome, Jesús Resendiz-Sanchez, Rafael Franco-Cendejas, Patricia Rodriguez-Zulueta, Dora E. Corzo-León

**Affiliations:** 1 Department of Infectious Diseases and Hospital Epidemiology, Hospital General Dr Manuel Gea González, Mexico City, Mexico; 2 Laboratory of Microbiology and Department of Infectious Diseases, Instituto Nacional de Rehabilitación, Mexico City, Mexico; 3 Laboratory of Mycology, Hospital Infantil de México, Mexico City, Mexico; 4 Aberdeen Fungal Group. MRC Centre of Medical Mycology, Wellcome Trust Strategy Award. Institute of Medical Sciences, University of Aberdeen, Aberdeen City, Scotland, UK

**Keywords:** Mucormycosis, Diabetes, Proliferation, Germination, *Rhizopus oryzae*

## Abstract

Metabolic control improves outcomes associated with mucormycosis. The aim of this study was to compare the *in vitro* proliferation of *Rhizopus oryzae* in blood of individuals with and without diabetes at different glycaemic levels. Ninety-five individuals were included. Blood samples from each participant were incubated with sporangiospores of *R. oryzae*. The germination, filamentation and growth of *R. oryzae* were compared at different time points. Four groups were defined, one without (group A, *n* = 30) and three with diabetes: group B (HbA1c ≤7%, *N* = 24), group C (HbA1c 7.1–9%, *N* = 20) and group D (HbA1c > 9%, *N* = 21). The percentage of germinated sporangiospores was higher in the group A after 6 h (group A 56% ± 3, group B 35% ± 4, group C 48% ± 4, group D 46% ± 1.4, *p* = 0.01), 12 h (group A 54% ± 1.4, group B 19% ± 4, group C 16% ± 1, group D 9.5% ± 5, *p* < 0.001) and 24 h (group A 29% ± 1, group B 12% ± 4, group C 13.5% ± 3.5, group D 12% ± 1, *p* < 0.01). The filamentation was higher in groups with diabetes. Group B showed higher filamentation grade than group A at 6 h (0.4 ± 0.04 vs 1 ± 0.09, *p* < 0.001) and 24 h (1.6 ± 0.05 vs 2.1 ± 0.1, *p* = 0.05). In conclusion, *R. oryzae* proliferation was higher among diabetic individuals, including good glycaemic control, than among non-diabetic individuals.

**Electronic supplementary material:**

The online version of this article (doi:10.1007/s11046-017-0174-0) contains supplementary material, which is available to authorized users.

## Introduction

The principal agent causing mucormycosis is *Rhizopus oryzae*. Mucormycosis is a lethal fungal infection affecting mainly individuals with diabetes, individuals with haematological malignancies [1–4] and individuals with trauma [5, 6]. Chronic and acute forms of glycaemic uncontrolled diabetes have been described as main risk factors in the development of mucormycosis [7–10], and this is due to the role of glucose in modulating angioinvasion [11]. Metabolic control has shown to decrease the susceptibility to mucormycosis and to improve the survival rate associated with this infection in animal models [12, 13]. Also, it has been highlighted, by clinical guidelines and experts in the field, that metabolic control is an essential component in the management of mucormycosis when it affects diabetic populations [14, 15]. In general, one of the recommendations in metabolic control of diabetes is to get a target of glycosylated haemoglobin (HbA1c) <7% [16]. Hence, the aim of this study was to compare the *in vitro* proliferation *of R. oryzae* in blood of individuals with and without diabetes. Our results showed lesser and slower *in vitro* proliferation of *R. oryzae* in samples of diabetic individuals and good glycaemic control in comparison with uncontrolled counterparts; however, it was still higher than the individuals without diabetes.

## Materials and Methods

### Included Population and Blood Samples

**Fig. 1:**
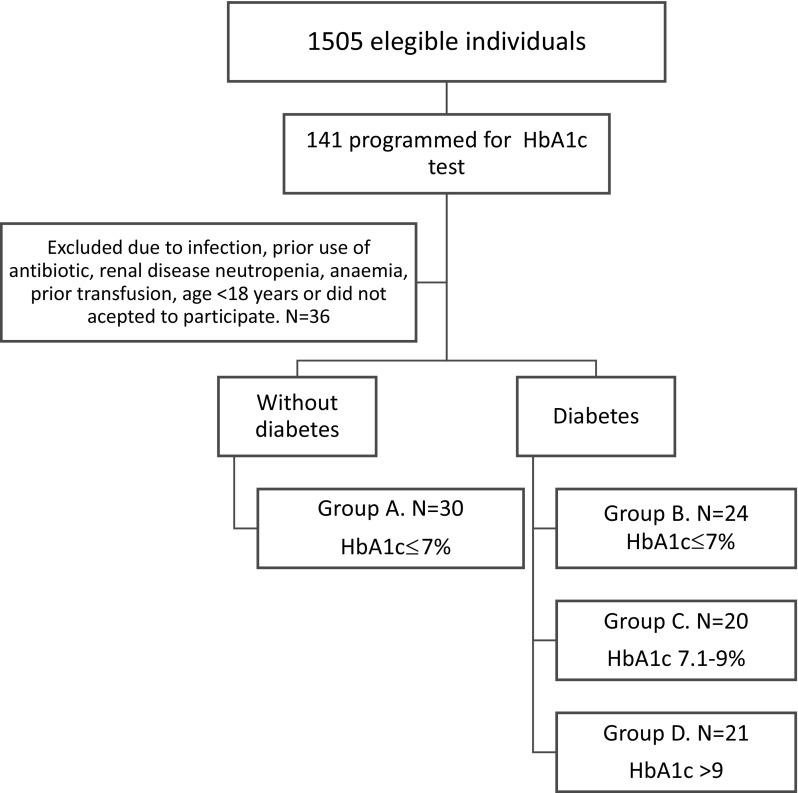
Study design and categories of the study groups. The selection of the participants was done based on previously check-up appointments, and they had to have a HbA1c test programmed. The study groups were categorised depending on the HbA1c levels,* group A*: individuals without diabetes, individuals with diabetes were* group B* (HbA1c ≤7%),* group C* (HbA1c 7.1–9%),* group D* (HbA1c > 9%)

This project was approved by the local IRB at Hospital General Dr Manuel Gea González with the number 36-55-2015. The selection of participants was based on their previously programmed hospital check-up appointments and the period of time when this study was held. During this period, 1505 individuals were identified as potential participants but only 141 had programed an appointment for HbA1c test. Finally, 95 individuals were included, and blood samples and clinical data were obtained. After a revision of medical records to confirm the diabetes status of the selected individuals (the diagnosis of diabetes was done independently by the healthcare provider/physician as part of a regular standard of care), they were categorised into groups with and without diabetes and by HbA1c level, as shown in Fig. 1.

One blood sample was drawn from each participant in tube without anticoagulant (SST BD Vacutainer^®^). All samples were subject to mechanical and freezing cellular lysis. Mechanical cellular lysis consisted of vortexing the sample for 5 min; meanwhile, freezing cellular lysis consisted of storing the samples at −70 °C until their use for further *in vitro* assays.

### Strains and Culture

During the *in vitro* assays, a respiratory clinical isolate of *R. oryzae* was used. This isolate was identified by conventional methods and sequencing. Conventional methods consisted of morphology identification of colony and microscopy using lactophenol cotton blue stain. ITS1-2 regions were amplified and sequenced using the universal primers ITS1/ITS4 previously published elsewhere [17], nucleotide sequence accession number MF379466.

The *R. oryzae* strain was cultured and maintained in Sabouraud dextrose agar (SDA) for five days at 30 °C. Following the CLSI M38A guidelines, sporangiospores suspension was collected to obtain a solution at 0.5 McFarland corresponding to 10^5^ sporangiospores/mL. Ninety-five sporangiospores suspensions from the same strain, one for each participant, were collected. Sporangiospores suspensions were kept frozen at −70 °C until their further use.

### Design of the Assay

For *in vitro* interaction assays, after thawing each blood sample and sporangiospores, 500 μl of blood of each participant and 500 μl of sporangiospores suspension (a dilution 1:2) were mixed and incubated at 30 °C. Each assay was performed in duplicate.

### Definitions

**Fig. 2:**
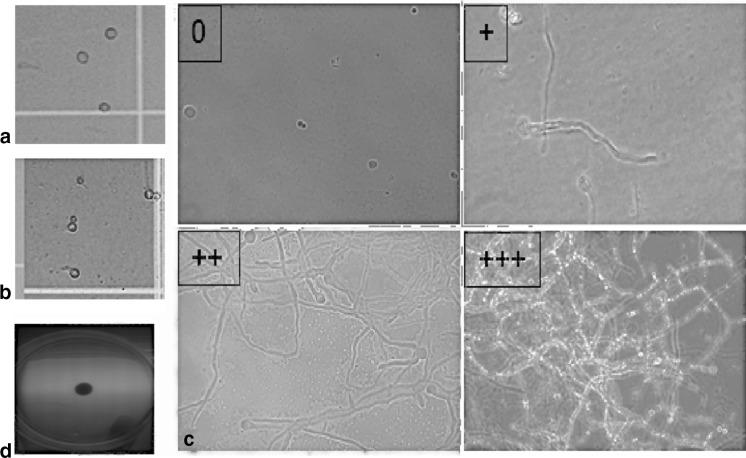
Definitions of the variables used to evaluate *in vitro* growth of *R. oryzae* in this study. **a** Number of sporangiospores per mL (Magnification ×40), **b** germination rate (elongated bodies per total of sporangiospores seen per mL, magnification ×40), **c** grade of filamentation/hypha formation per 100 fields (0 = no hyphae in 100 fields, + = 1–100 hyphae in 100 fields, ++ = 101–200 hyphae in 100 fields, +++ = more than 201 hyphae in 100 fields); magnification ×240, **d** growth in SDA measured in Mm. Sporangiospores and germinated bodies were counted using an haemocytometer

The growth of *R. oryzae* was evaluated at the time of the inoculation (time 0), and at 3, 6, 12 and 24 h of incubation. Observations were made in the same way for each one of the 95 samples. These observations were as follows: 1) number of sporangiospores and germination rate per mL, 2) filamentation/hyphae formation and 3) growth in Sabouraud dextrose agar (SDA). The number of sporangiospores was estimated per mL using a haemocytometer, and germination rate was estimated dividing the elongated sporangiospores by the total number of sporangiospores. To evaluate the filamentation, 10 μl of the sample on slide with 40% KOH solution was observed using light microscopy at 40× objective. The grade of filamentation was evaluated as follows: 1) Grade 1: 1–100 hyphae observed per 100 fields, 2) Grade 2: 101–200 hyphae per 100 observed fields and 3) Grade 3: >200 hyphae per 100 observed fields. SDA growth was measured in millimetres after culturing 10 μl of each sample in a SDA after 12 h of incubation, as shown in Fig. 2.

### Statistical Analysis

Results are presented as proportions and/or median or mean values (mean corresponding to the duplicated assays) as required. Comparisons were made with Kruskal–Wallis, one-way ANOVA, Fisher’s LSD and Dunnett’s tests as appropriate. *P* value ≤ 0.05 was considered statistically significant when two groups were compared and *p* value ≤ 0.01 when four groups were compared between them. Statistical tests were performed using SPSS Inc 24 software (IBM Corporation, New York, USA).

## Results

The 95 individuals included in this study were categorised depending on the HbA1c level. The categorisation was made after all *in vitro* assays were performed to be blind during the assay.

### Clinical Characteristics Between Groups were not Statistically Different Except for Glycaemic Control

Age, statin use, biochemical parameters (such as creatinine levels and haematic cytometry), except fasting glucose and HbA1c, were not significantly different between groups as given in Table 1. Only fasting glucose and HbA1c were different between groups (*p* < 0.05). The highest fasting glucose value was 456 g/dL and was found in one subject in group D. Acute hyperosmolar state was ruled out in this subject. Levels of HbA1c were ≤7% for groups A and B (median, minimum–maximum values 5.7, 4–6.4% vs 6.45, 5.3–7%) and for group C and group D > 7.1 (median, minimum–maximum values 8, 7.1–8.9% vs 10.8, 9.3–15.3%).

**Table 1:** Clinical characteristics of the population included in this study

Characteristics	Group A^1^ *n* = 30 (%)	Group B^1^ *n* = 24 (%)	Group C^1^ *n* = 20 (%)	Group D^1^ *n* = 21 (%)
Gender (male)	21 (70)	16 (67)	13 (65)	13 (62)
Age (years)	50 (26–81)	55 (30–92)	62 (21–88)	59 (30–85)
Prior use of statins	5 (17)	6 (25)	8 (40)	6 (27)
Obesity	4 (13)	1 (4)	1 (5)	1 (5)
Hypertension	6 (20)	7 (29)	7 (35)	4 (19)
Dyslipidaemia	3 (10)	1 (4)	1 (5)	0 (0)
Hypothyroidism	6 (20)	4 (17)	0 (0)	1 (5)
Fasting glucose (g/dL)*	94 (79–112)	108 (69–153)	154 (94–239)	192 (83–456)
HbA1c (%) *	5.7 (4–6.4)	6.45 (5.3–7.0)	8 (7.1–8.9)	10.8 (9.3–15.3)
WBC (×10^3^/mm^3^)	7.3 (3–14.4)	8 (6.6–13.4)	7.9 (5.4–12.5)	7 (4–10.1)
Haemoglobin (×10^3^/mm^3^)	14.8 (10–17)	14.05 (11–17)	14.7 (12–18)	14.6 (11–18)
MCV	90 (65–118)	89 (68–97)	89 (83–96)	89 (71–99)
MCH	31 (21–38)	30 (21–32)	30 (28–33)	30 (22–34)
Platelet count (×10^3^/mm^3^)	236 (145–411)	251 (110–405)	217 (162–335)	237 (116–470)
Creatinine (mg/dL)	0.7 (0.59–1.26)	0.69 (0.48–1.0)	0.87 (0.5301.3)	0.71(0.53–1.2)

^1^Group A (individuals without diabetes), Group B (individuals with DM2 and HbA1c < 7%), Group C (individuals with DM2 and HbA1c 7.1–9%), Group D (individuals with DM2 and HbA1c > 9%)

Results are presented as median, minimum and maximum values and percentages as required

*HbA1c* glycosylated haemoglobin, *WBC* white blood cells, *MCV* mean corpuscular volume, *MCH* median corpuscular haemoglobin

* *p* value < 0.05. Pearson’s X^2^ or Fisher’s tests were used as required and adjusted depending on the degrees of freedom in each case. For analysis of quantitative parameters, Kruskal–Wallis test was used

### *In vitro* Germination Process Started Earlier in all Groups with Diabetes but Lasts Longer in the Group Without Diabetes

**Fig. 3:**
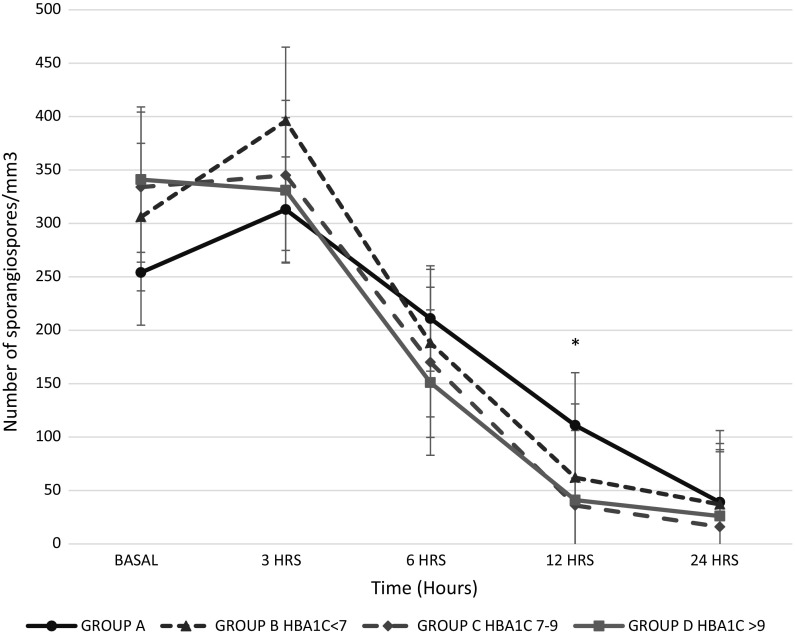
Number of sporangiospores during the period of observation. Plot showing the results presented as mean and standard deviation obtained from the duplicated experiments. *N* = 95. One-way ANOVA, *p < 0.01.* Group A*: individuals without diabetes.* Groups B* (HbA1c ≤7%),* C* (HbA1c = 7.1–9%) and* D* (HbA1c > 9%): individuals with diabetes. The decrease in number of sporangiospores corresponds to the start of the germination process

**Fig. 4:**
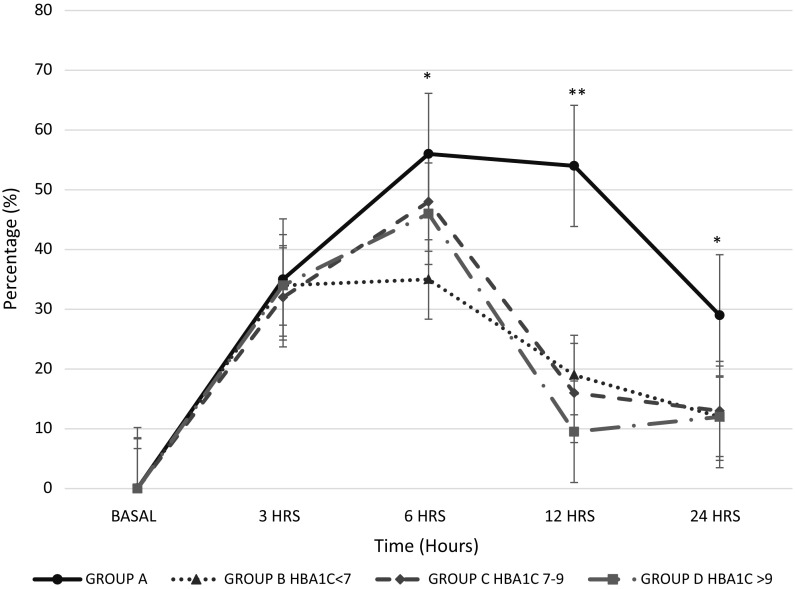
Germination rate during the period of observation. Plot showing the results presented as mean and standard deviation obtained from the duplicated experiments. *N* = 95, One-way ANOVA, *p < 0.01, **p < 0.001.* Group A*: individuals without diabetes.* Groups B* (HbA1c ≤7%),* C* (HbA1c = 7.1–9%) and* D* (HbA1c > 9%): individuals with diabetes. Decreasing the germination rate corresponds to the start of the filamentation process

The number of sporangiospores at the beginning of the assay between groups (254 vs 306 vs 334 vs 341, *p* = 0.21) decreased after 3 h of incubation (Fig. 3). Differences in the decreasing number of sporangiospores were evident between the group without diabetes compared with the groups with diabetes after 6 h of incubation (group A 111 ± 2.1 vs group B 62 ± 5.6, group C 36 ± 9.8, group D 41 ± 5, *p* < 0.001). In groups C and D with diabetes, levels of HbA1c between 7.1–9% and >9%, respectively, the number of sporangiospores did not differ between them (group C 36 ± 9.8 vs group D 41 ± 5, *p* = 0.8); however, the number of sporangiospores in these groups decreased higher and faster than in the group B with HbA1c level in glycaemic control (HbA1c < 7%). After 6 h of incubation, between groups A (without diabetes) and B, the decrease in sporangiospores was statistically different (group A 111 ± 2.1 vs group B 62 ± 5.6, *p* = 0.005) even when both groups had levels of HbA1c < 7%. It was evident that the decreasing number of sporangiospores corresponded to the starting of the germination process. The percentage of germinated sporangiospores was higher in the group A (without diabetes) after 6 h (group A 56% ± 3 vs group B 35% ± 4, group C 48% ± 4, group D 46% ± 1.4, *p* = 0.01), 12 h (group A 54% ± 1.4, group B 19% ± 4, group C 16% ± 1, group D 9.5% ± 5, *p* < 0.001) of incubation and was maintained until 24 h (group A 29% ± 1, group B 12% ± 4, group C 13.5% ± 3.5, group D 12% ± 1, *p* < 0.01), as shown in Fig. 4. The more evident germination difference between groups with and without diabetes was at 12 h of incubation, as shown in Table S1. The lower germination rate in groups with diabetes was due to the faster hyphae formation.

### The Grade of Filamentation *in vitro* of *R. oryzae* was Higher in all Groups with Diabetes Including Those with Glycaemic Control when Compared with the Group Without Diabetes

**Fig. 5:**
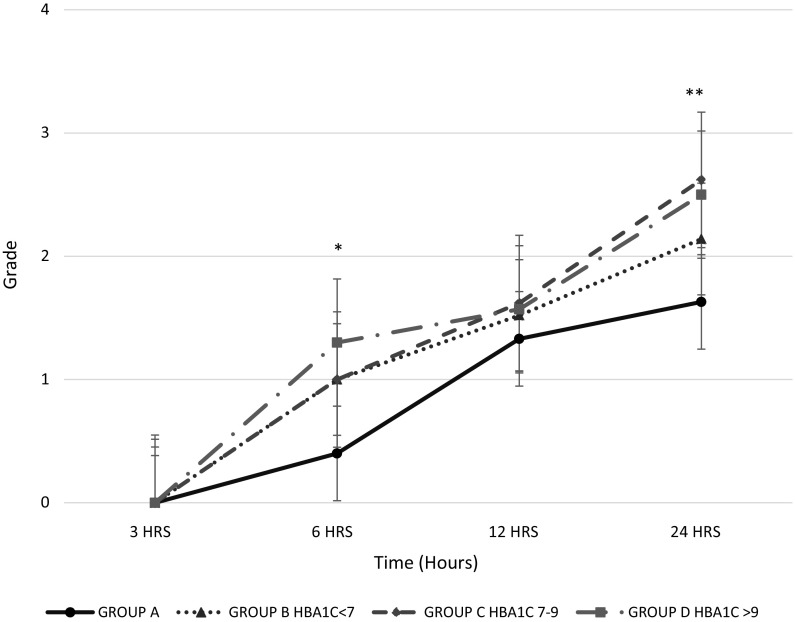
The grade of filamentation during the period of observation. Plot showing results presented as mean and standard deviation obtained from the duplicated experiments. *N* = 95. One-way ANOVA, *p < 0.001, ** *p* = 0.005. Grade of filamentation/hypha formation per 100 fields (0 = no hyphae in 100 fields, + = 1–100 hyphae in 100 fields, ++ = 101–200 hyphae in 100 fields, +++ = more than 201 hyphae in 100 fields).* Group A*: individuals without diabetes.* Groups B* (HbA1c ≤7%),* C* (HbA1c = 7.1–9%) and* D* (HbA1c > 9%): individuals with diabetes

The grade of filamentation was higher and faster in all groups with diabetes compared with the group without diabetes after 3 h of incubation (Fig. 4). The group B (with diabetes and HbA1c ≤7%) showed higher and faster grade of filamentation than individuals without diabetes at 6 h (group A 0.4 ± 0.04 vs group B 1 ± 0.09, *p* < 0.001) and 24 h (group A 1.6 ± 0.05 vs group B 2.1 ± 0.1, *p* < 0.05) (Fig. 5). All groups with diabetes had higher filamentation grade than the group A, as shown in Table S2. Between all the diabetes groups, the grade of filamentation was higher for groups B and C, as shown in Table S3. Also, when mycelial growth was evaluated in SDA, groups with diabetes initiated the growth faster than the group without diabetes at 3 h (group A 1 mm ± 0.14, group B 3 mm ± 0.09, group C 2.3 mm ± 0.17, group D 2.23 mm ± 0.13, *p* < 0.001). After 3 h of incubation on SDA, the growing was similar between groups being the group D the one with higher and faster growing out of all the other groups (Table 2).

**Table 2:** The growth of *R. oryzae* in Sabouraud dextrose agar

Group (*N* = 95)	3 h (mm)*	6 h (mm)**	12 h (mm)***	24 h (mm)
A (*n* = 30)	1.0 ± 0.14	7.8 ± 0.7	34 ± 0.33	82 ± 0.7
B (*n* = 24)	3.0 ± 0.09	8.7 ± 0.8	30 ± 2.5	81 ± 2.1
C (*n* = 20)	2.3 ± 0.17	9.4 ± 0.2	32 ± 0.56	83 ± 0.7
D (*n* = 21)	2.2 ± 0.13	11.0 ± 0.8	34 ± 0.14	81 ± 1.4

The results are presented as mean and standard deviation obtained from the duplicated experiments. Group A: individuals without diabetes. Group B (HbA1c ≤7%), C (HbA1c = 7.1–9%) and D (HbA1c > 9%) are individuals with diabetes. Mm = millimetres. *P* values shown in this table were calculated with Fisher’s LSD test

A versus B, both groups had HbA1c < 7%, but group A had not diabetes and group B had diabetes, * *p* = 0.0001, *** *p* = 0.03

B versus C, groups with diabetes with HbA1c < 7% versus 7.1–9%, * *p* = 0.005

B versus D, groups with diabetes with HbA1c < 7% versus > 9%, * *p* = 0.003, **p = 0.02, *** 0.04

C versus D, groups with diabetes with HbA1c 7–9% versus > 9%, *p* = NS

Finally, to rule out if the *R. oryzae* growth was influenced by the use of statins, a statistical analysis of all the growing characteristics was performed and no differences were found (Table S4). On the other hand, it was noted that *R. oryzae* germination and filamentation were higher when fasting glucose levels were higher than 200 g/dL (Table S5), and these levels were found only in groups C (HbA1c levels between 7.1 and 9%) and D (HbA1c > 9%) with diabetes (Fig. 5).

## Discussion

This study describes that the beginning of filamentation process in *R. oryzae* depends on the length of germination process. In samples of the group without diabetes, germination period was longer, reflecting that the hyphae were developed after a longer period; meanwhile, filamentation process started earlier in blood samples of groups with uncontrolled diabetes. Interestingly, in the controlled diabetic group, the filamentation grade was lower than in the uncontrolled diabetic groups but significantly higher when compared with the non-diabetes group.

The higher growth capacity of *R. oryzae* in samples of individuals with diabetes could be explained by high concentrations of glucose and iron being the main factors favouring the germination and fungal invasion of *R. oryzae* [10, 18]. Iron exists in two states, Fe^2+^ (ferrous) or Fe^3+^ (ferric), and the latest state is insoluble which limits its transport to the fungal intracellular compartment [18]. Iron is essential for *R. oryzae* growth, this pathogen has developed mechanisms to improve the assimilation of iron such as ferritins to store it, internal and external siderophores, and reductase/permease systems to improve iron uptake, and also iron can be obtained by *R. oryzae* from host haemoglobin [19]. In individuals with diabetic ketoacidosis, it is known that low pH promotes the release of iron [13]; however, hyperglycaemia, by itself, also promotes the damage of iron-sequestering proteins such as haemoglobin causing the releasing of serum-free iron [20]. In this study, none of the individuals had diabetic ketoacidosis although individuals with high HbA1c had also higher fasting glucose levels, meaning the main factor for the higher fungal growing was due to high levels of glucose, and probably the secondary releasing of iron due to hyperglycaemia.

The *GRP78* protein has been previously reported as the receptor for CotH3 ligand in *R. oryzae* [21], allowing the interactions between the host and fungi. The expression of *GRP78* protein is promoted by high concentrations of glucose, iron and acidotic environments [18]. The overexpression of *GRP78* protein leads to higher rates of endocytosis and cellular damage allowing the invasion, injury and dissemination of *R. oryzae.* In our study, the cells were lysed and therefore it could not be said that the higher concentration of GRP78 protein is playing a role in the higher growth rates of *R. oryzae* in diabetic groups.

The main observation in this study was that despite the glycaemic control, diabetic population could still be at risk of mucormycosis and, hence, have worst clinical outcomes. Tuberculosis is one example of disease where glycaemic control decreases the rates of infection but not at non-diabetic population rates. The incidence of tuberculosis could be twice higher in individuals with controlled diabetes compared with individuals without diabetes [22]. Similar observations have been also reported for urinary tract infections [23] and major macrovascular complications [24].

On the other hand, the role of HbA1c as the best marker of glycaemic control has been debated because it correlates with chronic sustained hyperglycaemia but not with all the acute glucose variability such as postprandial hyperglycaemia excursions which are best evaluated with mean amplitude of glycaemic excursion [25]. This implies that individuals with controlled diabetes in our study, although having normal fasting glucose levels, could be experiencing hyperglycaemic postprandial periods in a regular basis, which are not experienced by non-diabetic individuals. These hyperglycaemic episodes could be having indirect effects on releasing of free serum iron, and therefore, this mechanism would be favouring the *R. oryzae* growth in our assays. Unfortunately, the measurement of free iron was not taken in our study due to its original design. The impact of this glycaemic variability has been previously associated with higher risk of sepsis and mortality in individuals suffering burns and autologous haematopoietic cell transplant recipients [26, 27].

The findings in this study require to be studied and described in greater depth. In the future, interactions with host will be evaluated by our group using *in vitro* and *in vivo* models. At the authors’ knowledge, this is the first study reporting individuals with diabetes and good glycaemic control could still be at higher risk of mucormycosis than the non-diabetic individuals. These findings could partially answer and lead to further research of why more than 50% of the mucormycosis cases are presented without diabetic ketoacidosis [2] and why some cases have chronic evolution, instead of acute presentation [28], without an apparently associated factor with this clinical feature.

## Conclusion

Individuals with diabetes and good glycaemic control had lesser and slower *in vitro* proliferation of *R. oryzae* compared with uncontrolled ones; however, it was still higher than the individuals without diabetes. The main observation in this study was that despite the glycaemic control, diabetic population might have a higher risk of developing mucormycosis.

## Electronic Supplementary Material

Below is the link to the electronic supplementary material.

Supplementary material 1 (DOCX 78 kb)
