# Supplementary material for: Higher In vitro Proliferation Rate of Rhizopus oryzae in Blood of Diabetic Individuals in Chronic Glycaemic Control Compared with Non-diabetic Individuals
Source: Mycopathologia. 2017 Jul 6;182(11):1005–14. doi: 10.1007/s11046-017-0174-0 (PMC5684257; doi:10.1007/s11046-017-0174-0)
Supplement: Supplementary file 1 — Supplementary material 1 (DOCX 78 kb) [file 11046_2017_174_MOESM1_ESM.docx]

**Supplementary Table 1**

**p Values of germination rates when groups with and without diabetes are compared at different time points.**

| Germination rate | Adjusted p value* | | | |
| --- | --- | --- | --- | --- |
| Group comparison** | at 3 hrs | At 6 hrs | At 12 hrs | At 24 hrs |
| A-B | 0.94 | 0.008 | 0.001 | 0.009 |
| A-C | 0.69 | 0.16 | 0.0009 | 0.01 |
| A-D | 0.97 | 0.09 | 0.0005 | 0.009 |

**Control group was group A without diabetes

*Adjusted p Value calculated with Dunnett test

**Supplementary Table 2**

**p Values of filamentation grade when groups with and without diabetes are compared at different time points.**

| Filamentation grade | Adjusted p value* | | |
| --- | --- | --- | --- |
| Group comparison** | At 6 hrs | At 12 hrs | At 24 hrs |
| A-B | 0.0006 | 0.23 | 0.04 |
| A-C | 0.0008 | 0.08 | 0.004 |
| A-D | 0.0002 | 0.05 | 0.007 |

**Control group was group A without diabetes *Adjusted p Value calculated with Dunnett test

**Supplementary Table 3.**

**Differences in filamentation grade between groups.**

| Filamentation grade | Means | | |
| --- | --- | --- | --- |
| Group | At 6 hrs | At 12 hrs | At 24 hrs |
| A | 0.4^a^ | 1.3^a^ | 1.6^a^ |
| B | 1.06 ^b^ | 1.5 ^a,b^ | 2.2 ^b^ |
| C | 1 ^b^ | 1.6 ^b^ | 2.6 ^c^ |
| D | 1.26^c^ | 1.7^b^ | 2.4 ^b,c^ |

Groups sharing a letter are not significantly different

Fisher’s LSD Method

**Supplementary table 4.**

**Parameters of *R. oryzae* growth between individuals on statins compared with those not on statins**

|  | Statins use | | |
| --- | --- | --- | --- |
| Growth parameter | YES N=25 | NO N=70 | p value |
| Sporangiospores 3 hrs (#) | 274 (515) | 318(1057) | 0.2 |
| Sporangiospores 6 hrs (#) | 131(341) | 174(569) | 0.055 |
| Sporangiospores 12 hrs (#) | 33(185) | 46(377) | 0.22 |
| Sporangiospores 24 hrs (#) | 21 (63) | 25 (206) | 0.3 |
| Germination basal (%) | 0 (1.15) | 0 (1.15) | 0.46 |
| Germination 3 hrs (%) | 32 (181) | 32 (179) | 0.36 |
| Germination 6 hrs (%) | 42(65) | 45(75) | 0.35 |
| Germination 12 hrs (%) | 16 (98) | 17 (98) | 0.35 |
| Germination 24 hrs (%) | 16 (57) | 15(58) | 0.45 |
| Filamentation 6 hrs (Grade) | 1 (2) | 1 (2) | 0.6 |
| Filamentation 12 hrs (Grade) | 1.5 (2) | 1.5 (3) | 0.51 |
| Filamentation 24 hrs (Grade) | 2.5 (2.5) | 2 (3) | 0.25 |
| SDA growth 3 hrs (mm) | 1.5 (7) | 0.75 (10) | 0.27 |
| SDA growth 6 hrs (mm) | 7.5 (22) | 9 (21) | 0.94 |
| SDA growth 12 hrs (mm) | 32 (32) | 35 (68) | 0.233 |
| SDA growth 24 hrs (mm) | 85 (13) | 85 (23) | 0.76 |

Data are presented as median and range. Mann-Whitney Test.

**Supplementary Table 5**

**Parameters of *R. oryzae* growth between individuals with 2 different glucose levels**

|  | Fasting glucose level | | |
| --- | --- | --- | --- |
| Growth parameter | <200 g/dL  N=82 | ≥200 g/dL  N=12 | p value |
| Sporangiospores 3 hrs (#) | 306 (1058) | 340 (218) | 0.28 |
| Sporangiospores 6 hrs (#) | 161 (569) | 169 (330) | 0.65 |
| Sporangiospores 12 hrs (#) | 46 (377) | 23 (73) | 0.002 |
| Sporangiospores 24 hrs (#) | 25 (206) | 14 (55) | 0.052 |
| Germination basal (%) | 0 (1.15) | 0 | 0.38 |
| Germination 3 hrs (%) | 33 (181) | 29 (46) | 0.79 |
| Germination 6 hrs (%) | 41 (75) | 30 (69) | 0.3 |
| Germination 12 hrs (%) | 18 (98) | 2.6 (28) | 0.001 |
| Germination 24 hrs (%) | 16 (58) | 8 (24) | 0.257 |
| Filamentation 6 hrs (Grade) | 1 (2) | 1 (1) | 0.001 |
| Filamentation 12 hrs (Grade) | 1.5 (3) | 2 (3) | 0.003 |
| Filamentation 24 hrs (Grade) | 2 (3) | 2.75 (2) | 0.008 |
| SDA growth 3 hrs (mm) | 1 (10) | 1.5 (7) | 0.53 |
| SDA growth 6 hrs (mm) | 8 (22) | 11 (21) | 0.12 |
| SDA growth 12 hrs (mm) | 34 (39) | 35 (68) | 0.64 |
| SDA growth 24 hrs (mm) | 85 (23) | 84 (23) | 0.21 |

Data are presented as median and range. Mann-Whitney Test.
